# Zinc finger protein 598 inhibits cell survival by promoting UV-induced apoptosis

**DOI:** 10.18632/oncotarget.23643

**Published:** 2017-12-23

**Authors:** Qiaohong Yang, Romi Gupta

**Affiliations:** ^1^ Department of Pathology, Yale University School of Medicine, New Haven, CT 06510, USA

**Keywords:** zinc finger protein, UV-induced, apoptosis, upregulation, phenocopies

## Abstract

UV is one of the major causes of DNA damage induced apoptosis. However, cancer cells adopt alternative mechanisms to evade UV-induced apoptosis. To identify factors that protect cancer cells from UV-induced apoptosis, we performed a genome wide short-hairpin RNA (shRNA) screen, which identified Zinc finger protein 598 (ZNF598) as a key regulator of UV-induced apoptosis. Here, we show that UV irradiation transcriptionally upregulates ZNF598 expression. Additionally, ZNF598 knockdown in cancer cells inhibited UV-induced apoptosis. In our study, we observe that ELK1 mRNA level as well as phosphorylated ELK1 levels was up regulated upon UV irradiation, which was necessary for UV irradiation induced upregulation of ZNF598. Cells expressing ELK1 shRNA were also resistant to UV-induced apoptosis, and phenocopy ZNF598 knockdown. Upon further investigation, we found that ZNF598 knockdown inhibits UV-induced apoptotic gene expression, which matches with decrease in percentage of annexin V positive cell. Similarly, ectopic expression of ZNF598 promoted apoptotic gene expression and also increased annexin V positive cells. Collectively, these results demonstrate that ZNF598 is a UV irradiation regulated gene and its loss results in resistance to UV-induced apoptosis.

## INTRODUCTION

Ultraviolet irradiation induces DNA damage and consequentially apoptosis [1, 2]. Upon UV irradiation both replication and transcription machinery are affected [3–5]. Replication arrest resulting from UV-induced DNA damage leads to an active response in the form of a S phase checkpoint by which replication errors are avoided, that could otherwise lead to mutagenesis, chromosomal breakage and DNA recombination [6]. Notably, UV-induced DNA damage cause upregulation of several apoptotic genes and pathways that play critical roles in preventing malignant transformation [7]. Additionally, UV-induced apoptosis is also shown to be mediated by the upregulation of reactive oxygen species (ROS) and alteration of many signaling pathways like MAPK etc. [7–9]. However, there are other DNA damage-independent mechanisms that also lead to apoptosis upon UV irradiation, where apoptosis receptors are shown to play key role [3].

Cancer cell develop tolerance to UV-induced apoptosis by altering the expression of different genes and pathways [10, 11]. Studies have shown that human cells upon UV irradiation, upregulates DNA repair machinery that helps them to maintain genome integrity. In addition to this, cancer cells utilize various means to inhibit UV-induced apoptosis, like overexpressing certain cytokines [11], heat shock proteins [12], or anti apoptotic genes [13, 14] or by blocking death receptor activation [2, 15–17]. All these allows cancer cell survival even after UV irradiation-induced DNA damage. Since cancer cells adopt various means to evade UV-induced apoptosis and considering the possibility that they might be different in each cancer cell type our aim was to identify common genes and pathways that can sensitize multiple cancer cell types to UV-induced apoptosis and inhibit cancer cell growth. To find such candidate gene we performed a genome wide shRNA screen, which identified Zinc finger protein 598 (ZNF598) as a new regulator of UV-induced apoptosis. We find that ZNF598 expression is dependent on UV irradiation and its loss in different kind of cancer cells provides resistance to UV-induced apoptosis. We also show that transcription factor ELK1 regulates ZNF598 expression. ELK1 expression as well as its phosphorylation is increased upon UV irradiation and its knockdown, similar to ZNF598 knockdown, inhibits UV-induced apoptosis. Upon further investigation, we found that loss of ZNF598 inhibits UV-induced apoptotic genes and decreases percentage of annexin V positive cell as compared to control cell expressing nonspecific shRNA. In agreement with the role of ZNF598 in regulating the function of UV-induced apoptosis, we show that the ectopic expression of ZNF598 in cell leads to upregulation of apoptotic gene expression and also increase in annexin V positive cell as compared to control cell expressing vector. Collectively, we demonstrate that ZNF598 by regulating apoptotic gene expression functions as a new regulator of UV-induced apoptosis.

## RESULTS

### A genome wide shRNA screen identifies ZNF598 as a regulator of UV-induced apoptosis

**Figure 1:**
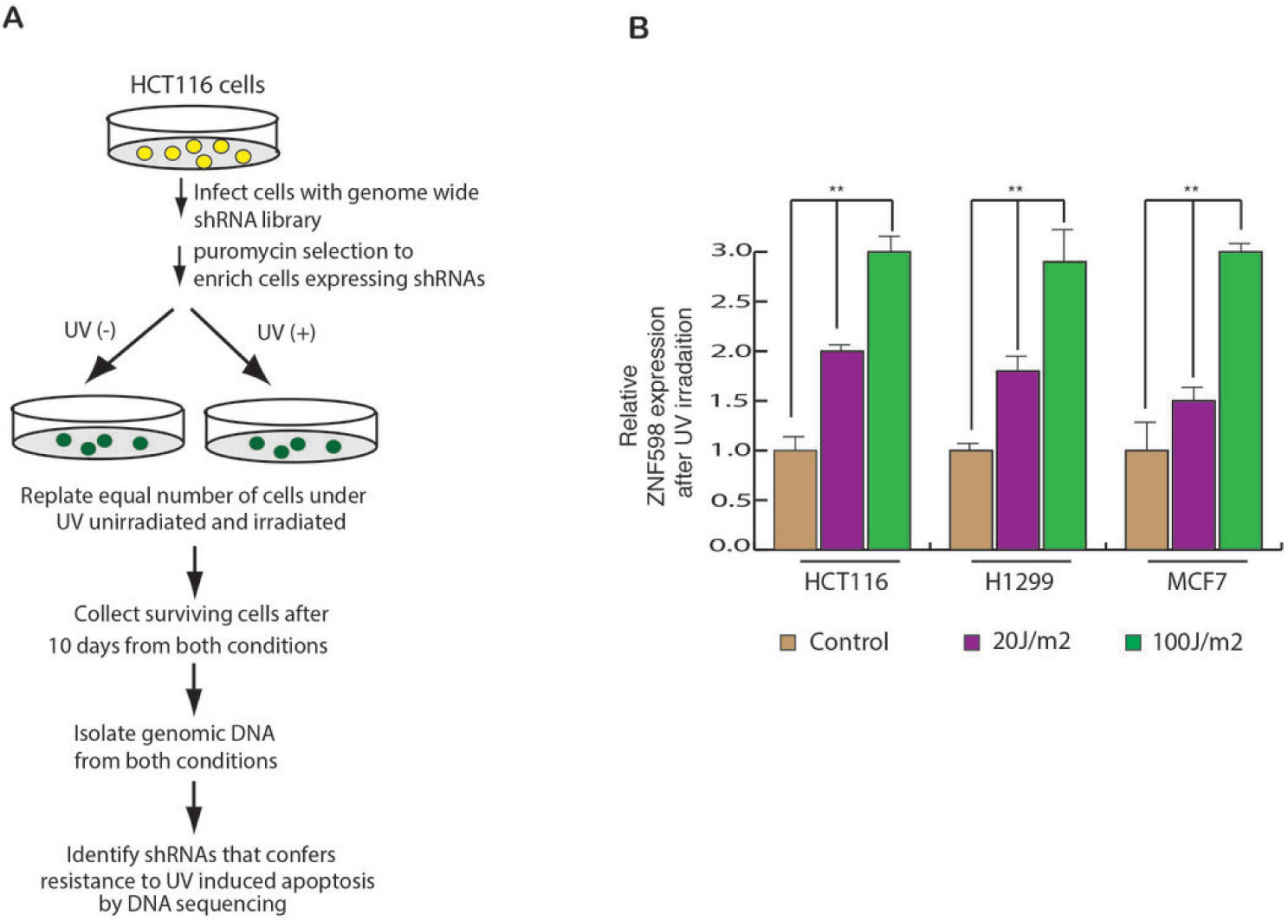
A genome wide shRNA screen identifies ZNF598 as regulator of UV-induced apoptosis (**A**) Schematics of the screen is described. It was performed in HCT116 cell line. (**B**) HCT116, H1299 and MCF7 cells were UV irradiated at 20 J/m^2^ and 100 J/m^2^ and gene expression was measured after 24 hours of irradiation. Relative ZNF598 expression is plotted with respect to control un-irradiated cell. Error bar shows Standard Error Mean (SEM). (^**^p < 0.001)

In order to identify the genes that regulate UV-induced apoptosis, we performed a genome-wide shRNA screen in HCT116 cells, using human lentiviral (TRC) shRNA libraries which consist of ∼85,000 lentiviral shRNA constructs. The library contains 4–5 shRNAs construct per gene, divided into 22 pools. To perform the shRNA screen, we infected the HCT116 cells with 22 pool of shRNA library. Infected HCT116 cells were puromycin selected to enrich the cells carrying shRNA. The selected cells were divided into two pools, where one pool was UV irradiated and the other pool was kept as control. The surviving cells were collected from both unirradiated as well as UV irradiated condition and genomic DNA was isolated and sequenced to identify the shRNA’s the were enriched in UV treated cells as compared to untreated cells. The shRNAs sequences were used to identify genes that are involved in UV-induced apoptosis (Figure 1A). The screen was set up similar to that used by a previous study, in which authors performed a genome wide RNAi screen to identify the genes required for resistance to ionizing radiation (IR) [18].

Among the list of identified shRNA, we found multiple shRNA enriched for gene RNF17, KLHL5 and ZNF598 (Table 1). However, after performing shRNA-mediated knockdown of these gene, we measured their UV sensitivity and found that only the cells expressing ZNF598 shRNAs showed significant resistance to UV-induced apoptosis (Supplementary Figure 1). This result led us to conclude that that loss of ZNF598 is important for promoting resistance to UV-induced apoptosis. Based on these observations we chose to study the role of ZNF598 in mediating UV-induced apoptosis.

**Table 1:** List showing shRNA of the genes identified in HCT116 after UV irradiation

Gene	shRNA sequence
Ring finger protein 148 (RNF148)	GCCATTACATCATGTATCTAT
Myosin regulatory light chain interacting protein (MYLIP)	CCCAATTAATAGGATAGCTTA
Ring finger protein 166 (RNF166)	ACCTTTGTGGACTACAGTATT
Mitochondrial E3 ubiquitin protein ligase 1 (MUL1)	AGCCCAAGAAGTGCCCTAT
Ring finger protein 181 (RNF181)	GCCCACTGACGACGACACTTA
Ring finger protein 17 (RNF17)^*^	CCATGCTCATTGAAAGACATT
Zinc finger, SWIM-type containing 2 (ZSWIM2)	TATTTGAATGTTGAATATAAA
Zinc finger protein 598 (ZNF598)^*^	CCTCGACAAATGGTCCTGTAA
TNF receptor-associated factor 3 (TRAF3)	CCTTCATTTACAGNGAGNGAT
Listerin E3 ubiquitin protein ligase 1 (LTN1)	CCTGTAAATCTAATCAGTGAA
Tripartite motif containing 34 (TRIM34)	GCCTTCCCATTTATCCATGTT
TNF receptor-associated factor 4 (TRAF4)	CGAAACTATGTGCGGGATGAT
Kelch-like 5 (Drosophila) (KLHL5)^*^	CCCTTAATCATGCCGAGCAAA
Neural precursor cell expressed, developmentally down-regulated 4-like (NEDD4L)	NCGGATGAGAATAGAGAACTT
HECT, C2 and WW domain containing E3 ubiquitin protein ligase 2 (HECW2)	GCCCAAACATTTCTTTGAGAT
Zinc finger and BTB domain containing 26 (ZBTB26)	CCTAACATTGACTTATGTCTT

(^*^) Multiple sequences.

Next, to measure the effect of UV irradiation on the expression of ZNF598, we irradiated multiple cancer cell lines with UV irradiation and measured ZNF598 mRNA level. We found that UV irradiation leads to elevated expression of ZNF598 in multiple cancer cell lines (Figure 1B). These results allowed us to conclude that ZNF598 is a UV regulated genes and its expression is transcriptionally upregulated as a result of UV irradiated.

### ZNF598 is necessary for protecting the cells from UV irradiation-induced apoptosis

**Figure 2:**
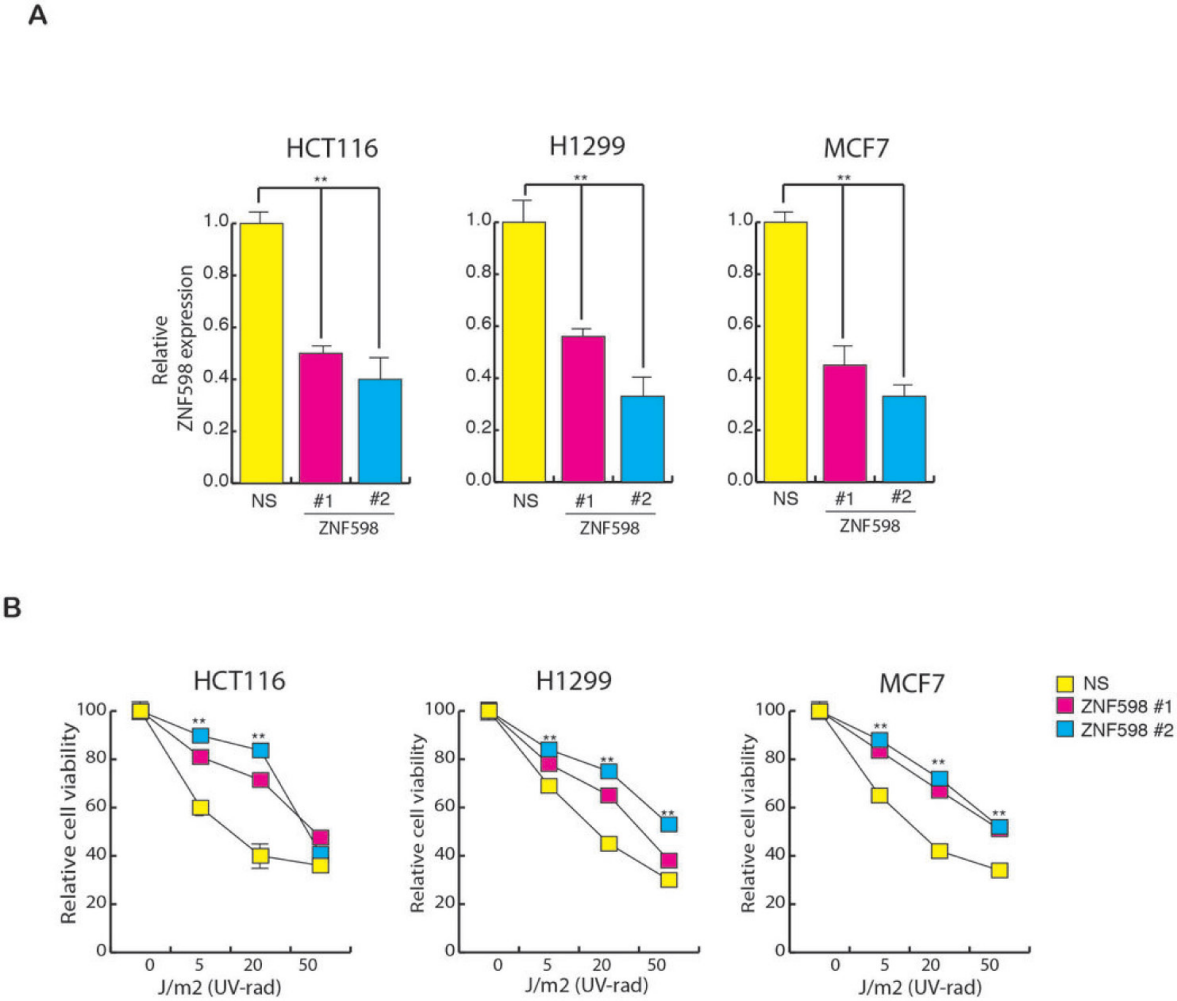
Loss of ZNF598 leads to resistance to UV-induced apoptosis (**A**) Relative ZNF598 expression in cell (HCT116, H1299 and MCF7) expressing NS shRNA or ZNF598 shRNA. Relative ZNF598 expression is plotted with respect to control cell expressing non-specific shRNA. (**B**) HCT116, H1299 and MCF7 cell expressing with non-specific shRNA or ZNF598 shRNA were UV irradiated at the indicated doses, and cell viability was measured by the trypan blue exclusion assay 48 hours post-irradiation. The cell viability relative to the un-irradiated control is plotted. Error bar shows Standard Error Mean (SEM). (^**^p < 0.001)

To further investigate whether ZNF598 plays a role in UV-induced apoptosis, we used short-hairpin RNAs (shRNAs) to knock down its expression in a variety of human cancer cell lines of different tissue origin (Figure 2A) and analyzed the sensitivity of these cells to UV irradiation. As shown in Figure 2B, the shRNA-mediated knockdown of ZNF598 in various human cell lines leads to resistance to UV-induced apoptosis. These results indicate that loss of ZNF598 leads to better survival of cells upon UV irradiation and hence its loss causes resistance to UV-induced apoptosis.

**Figure 3:**
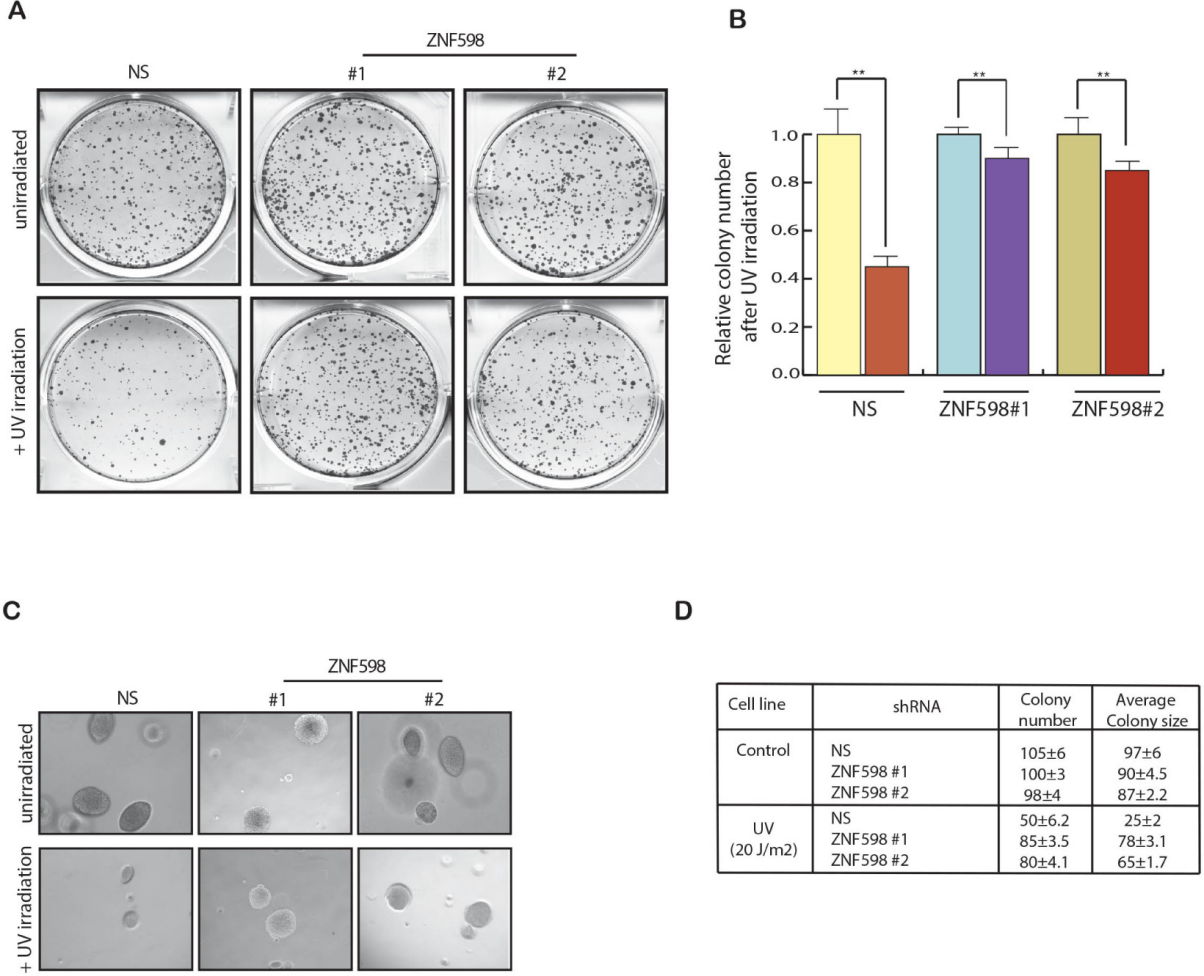
Loss of ZNF598 leads to increased cell survival upon UV irradiation (**A**) HCT116 cell lines stably expressing a non-specific (NS) shRNA or *ZNF598* shRNAs were analyzed for Clonogenic assay. To do so HCT116 cell lines expressing ZNF598 shRNAs or a non-silencing (NS) shRNA were UV irradiated 20 J/m^2^ and plated on 6 well plates along with control un-irradiated cells. Colonies were allowed to form and after 2 weeks the colonies were stained with Coomassie staining solution. (**B**) Number of colonies of each sample under control un-irradiated condition and also in also in UV irradiation condition was counted. Relative colony number is plotted with respect to control un-irradiated cell. HCT116 cell line expressing non-silencing (NS) or *ZNF598* shRNAs with un-irradiated of UV irradiated and were analyzed for their ability to grow in an anchorage-independent manner using soft agar assay. Representative soft-agar assay images are shown in left (**C**) and relative colony numbers and average colony size are shown in right (**D**). Error bar shows Standard Error Mean (SEM). (^**^p < 0.001).

Since we found that loss of ZNF598 cause resistance to UV-induced apoptosis, we further tested its effect on the cell growth first in clonogenic assay and then also in soft agar assay. Clonogenic is a long-term survival assay and is used to check the effect of a specific agent on the survival and proliferation of every individual cell in a population to form colony. To perform clonogenic assay, we UV irradiated the cells expressing ZNF598 shRNA along with control cells expressing non-specific shRNA and then measured their clone formation ability by plating equal number of cells to form colonies. In complete agreement with our short-term survival assays (Figure 2B), we find that the knockdown of *ZNF598* led to increase in colony number as compare to control cells expressing nonspecific shRNA upon UV irradiation suggesting its role in mediating resistance to UV-induced apoptosis (Figure 3A and 3B). To perform soft agar assay, which mimics *in vivo* growth in cell culture, we UV irradiated the cells expressing ZNF598 shRNA along with control cells expressing nonspecific shRNA and measured its growth in soft agar. As expected we found that cell expressing ZNF598 shRNA had larger number of colonies as well as bigger colon size as compared to control cells expressing non-specific shRNA upon UV irradiation (Figure 3C and 3D). These results indicate that loss of ZNF598 is essential for cancer cells to survive and proliferate upon UV irradiation.

Our results indicated that ZNF598 is UV responsive gene and its loss plays a role in mediating resistance to UV-induced apoptosis. Therefore, we also asked if any alteration in ZNF598 such as copy-number loss/inactivating somatic mutations are observed in human cancer samples. To answer that we analyzed the mutation and Copy number alteration data from cBioPortal. This analysis included 168 different studies and 48,511 cancer samples, including a large number of data generated by The Cancer Genome Consortium (TCGA). The result of this analysis is presented in the Supplementary Figure 2. Collectively, these analysis show that different cancer types have high frequency of mutations in ZNF598. This result allowed us to conclude that many cancer cells inactivate this gene to become resistant to UV-induced apoptosis.

### Transcription factor ELK1 is necessary for transcriptional up regulation of ZNF598

**Figure 4:**
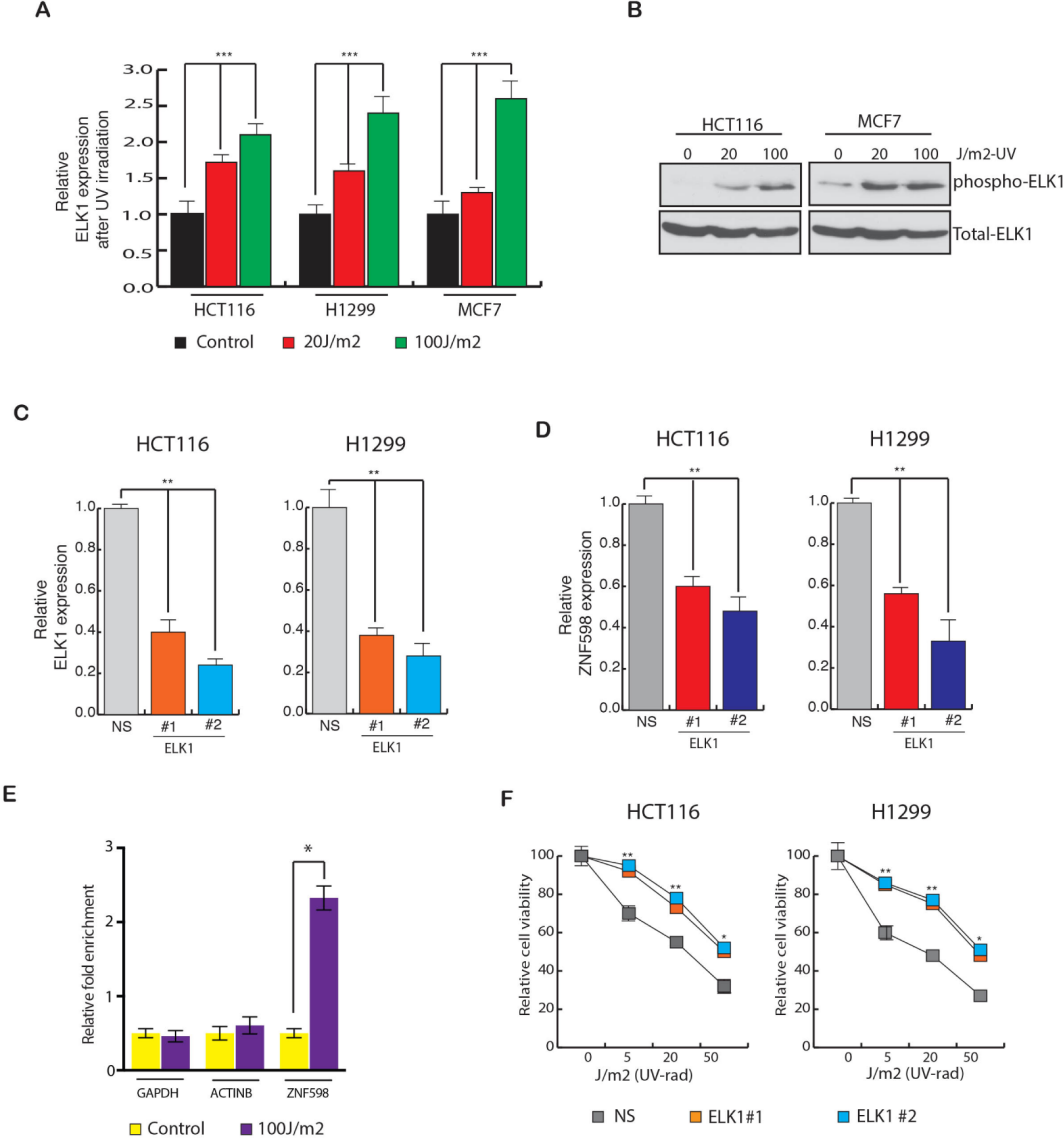
Transcription factor ELK1 stimulate ZNF598 expression (**A**) HCT116, H1299 and MCF7 cells were UV irradiated at 20 J/m^2^ and 100 J/m^2^ and gene expression was measured after 24 hours of irradiation. Relative ELK1 expression is plotted with respect to control un-irradiated cell. (**B**) Relative phospho and total ELK1 levels in HCT116 and MCF7 cells is measured after 24 hours of UV irradiation at 20 J/m^2^ and 100 J/m^2^ . (**C**) Relative ELK1 expression in cell (HCT116 and H1299) expressing NS shRNA of ELK1 shRNA. Relative ELK1 expression is plotted with respect to control cell expressing non-specific shRNA. (**D**) Relative ZNF598 expression in cell (HCT116 and H1299) expressing NS shRNA of ELK1 shRNA is shown. Relative ZNF598 expression is plotted with respect to control cell expressing non-specific shRNA. (**E**) HCT116 cells expressing non-silencing (NS) or *ZNF598* shRNAs were analyzed for ELK1 protein enrichment on *ZNF598* promoter using Chromatin immunoprecipitation (ChIP) assay. ACTINB and GAPDH promoter unrelated regions were used as negative controls. % Enrichment relative to input for indicated conditions for each promoter locus (ZNF598, ACTINB and GAPDH) is shown. (**F**) HCT116 and H1299 cell expressing with non-specific shRNA or ELK1 shRNA were UV irradiated at the indicated doses, and cell viability was measured by the trypan blue exclusion assay 48 hours post-irradiation. The cell viability relative to the un-irradiated control is plotted. Error bar shows Standard Error Mean (SEM). (^*^, ^**^ represents *p* < 0.01 and *p* < 0.001).

To determine the mechanism by which *ZNF598* transcription is up regulated in the cells, we analyzed the *ZNF598* promoter sequence using the transcription factor DNA-binding site-prediction programs PROMO and rVISTA 2. These analyses identified a conserved DNA-binding site for many transcription factors (Table 2). In order to understand which of them play role in regulating ZNF598 expression, we first measured the expression of these transcription factors upon UV irradiation (Supplementary Figure 3). Our result showed that out of the list of transcription factors that had conserved binding site on ZNF598 promoter, ELK1 levels changed significantly upon UV irradiation (Supplementary Figure 3 and Figure 4A). To also know if ELK1 phosphorylation too changes upon UV irradiation, we UV irradiated the cells and then measured p-ELK1 and total ELK1 levels. Our results showed that p-ELK1 levels too increase upon UV irradiation (Figure 4B).

**Table 2:** Factors predicted within a dissimilarity margin less or equal than 10% on ZNF598 Promoter region (2 KB)

Name of transcription factor (position on promoter)	Matrix width
T3R-beta1 [T00851]	9
AP-1 [T00029]	9
USF2 [T00878]	10
SRY [T00997]	9
GCF [T00320]	9
VDR [T00885]	9
NF-AT2 [T01945]	10
STAT1beta [T01573]	10
PPAR-alpha:RXR-alpha [T05221]	11
AhR:Arnt [T05394]	10
Ik-1 [T02702]	13
COUP-TF1 [T00149]	13
HOXD9 [T01424]	10
HOXD10 [T01425]	10
RAR-beta [T00721]	10
NF-AT1 [T01948]	10
CREB [T00163]	9
ATF-2 [T00167]	10
USF1 [T00874]	10
GATA-2 [T00308]	9
Sp1 [T00759]	10
Elk-1 [T00250]	9
ETF [T00270]	11
ATF-1 [T00968]	11
c-Ets-2 [T00113]	9
IRF-1 [T00423]	9
HIF-1 [T01609]	9
EBF [T05427]	11
AR [T00040]	9
MAZ [T00490]	13
POU2F2 (Oct-2.1) [T00646]	11
HNF-1C [T01951]	9
HNF-4alpha [T03828]	13
HNF-1B [T01950]	9

The list shows the transcription factors that have matric width of 9 or more nucleotide on promoter sequence.

Further to test the role of ELK1 in regulating of ZNF598 level, we knocked down ELK1 expression using shRNAs in different types of cancer cell (Figure 4C). In the ELK1 knockdown cells, ZNF598 expression was also measured. We observed that ELK1 knockdown resulted in reduced ZNF598 expression in multiple cancer cell lines (Figure 4D). These results indicated that ELK1 regulates the ZNF598 transcription. To also confirm that this correlation holds true in other public gene expression datasets, we analyzed oncomine datasets. Here, we identified many instances in which expression of ELK1 correlated with ZNF598 expression as shown in Supplementary Figure 4, confirming our finding that ELK1 and ZNF598 expression positively correlates and ELK1 regulates ZNF598 expression.

To determine if ELK1 is a direct regulator of *ZNF598* transcription and binds to *ZNF598* gene promoter sequence, we performed a chromatin immunoprecipitation (ChIP) assay. Our results show that ELK1 was significantly enriched on the *ZNF598* promoter sequence compared to the *ACTINB* or *GAPDH* promoter sequences upon UV irradiation (Figure 4E). Lastly, in order to know whether inhibition of ELK1 has similar effect on the cells upon UV irradiation as ZNF598 knockdown, we UV irradiated variety of cancer cell lines expressing *ELK1* shRNA to different UV doses and measured their survival ability. As shown in Figure 4F, we found that cell expressing ELK1 shRNA have increased survival upon UV irradiation as compared to control cells expressing non-specific shRNA and hence is involved in resistance to UV-induced apoptosis. These results in sum conclude that ELK1 regulates ZNF598 expression and phenocopy ZNF598 effect in cancer cells.

### Loss of ZNF598 inhibits apoptotic genes expression upon UV irradiation

**Figure 5:**
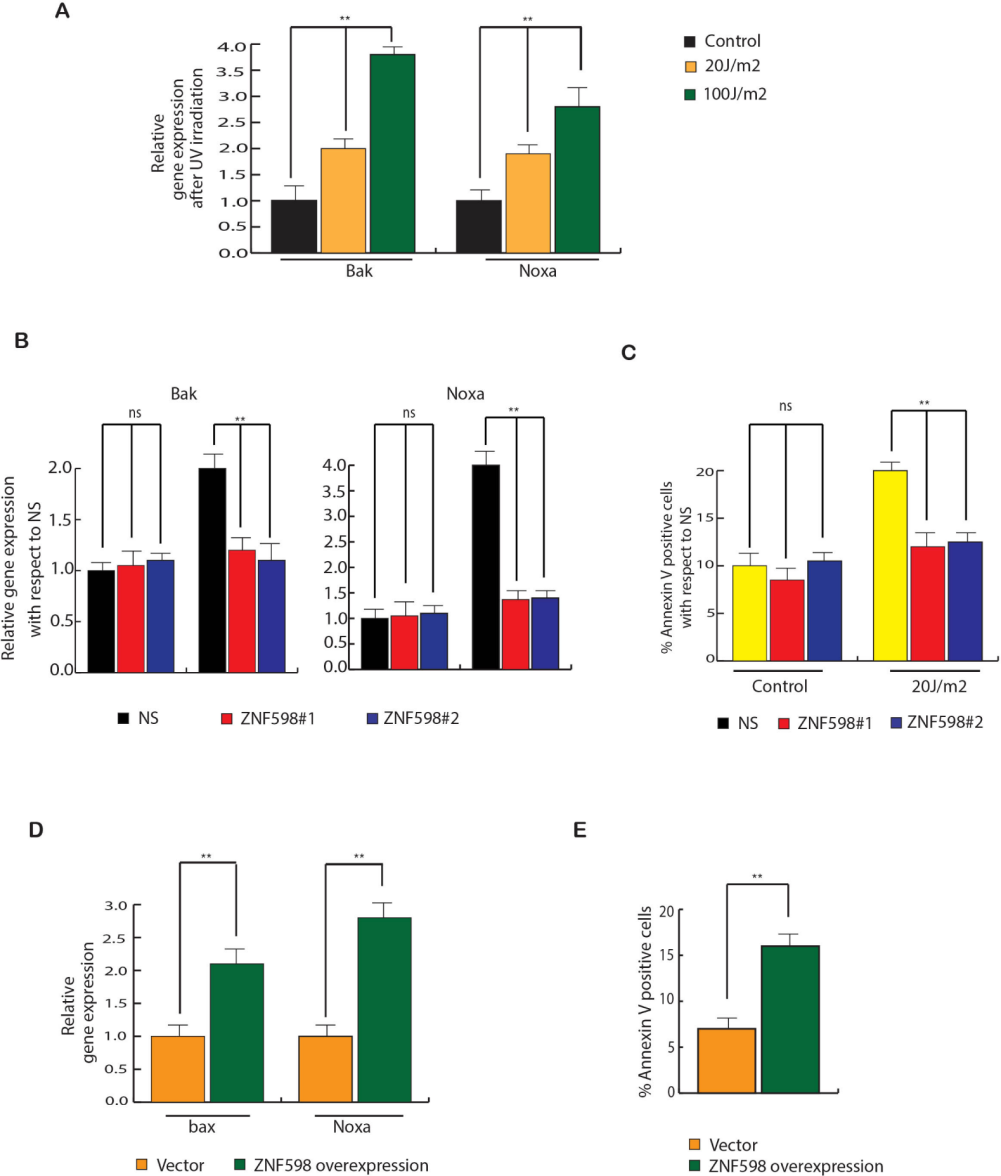
ZNF598 stimulates apoptotic gene expression upon UV irradiation (**A**) HCT116, H1299 and MCF7 cells were UV irradiated at 20 J/m^2^ and 100J/m^2^ and UV-induced apoptotic genes were measured after 24 hours of irradiation. Relative expression is plotted with respect to control un-irradiated cell. (**B**) HCT116 cells expressing non-specific or ZNF598 shRNA were UV irradiated at 100 J/m^2^ and UV-induced apoptotic genes were measured after 24 hours of irradiation. Relative expression is plotted with respect to control cells expressing non-specific both under irradiated and un-irradiated condition. (**C**) HCT116 cells expressing either non-silencing (NS) shRNA or *ZNF598* shRNAs were UV irradiated at different dose. Apoptotic cell death was measured after 48 hrs of UV irradiation. % Annexin V positive cells are plotted with respect to control cells expressing non-specific both under irradiated and un-irradiated condition. (**D**) ZNF598 or as a control vector was overexpressed in HCT116 and UV-induced apoptotic genes were measured. Relative gene expression is plotted with respect to control un-irradiated cell. (**E**) ZNF598 or as a control vector was overexpressed in HCT116 and apoptotic cell death was measured via annexin V staining. % Annexin V positive cells are plotted under indicated conditions. Error bar shows Standard Error Mean (SEM). (^**^p < 0.001), ns (not significant).

To determine the cause for decrease in apoptosis in cells expressing ZNF598 shRNA to UV irradiation as compared to control cell expressing non-specific shRNA, we examined the expression of various UV-induced apoptotic genes like bak and noxa. As a control, first we measured the expression of these apoptotic genes upon UV irradiation and as expected we found them to be increased upon irradiation (Figure 5A). Next, we measured UV-induced apoptotic genes in cell expressing ZNF598 shRNA and found that these cells expressed lower levels of UV-induced apoptotic genes as compared to control cell expressing non-specific shRNA upon irradiation (Figure 5B). This was consistent with apoptosis measured via Annexin V staining. Cells expressing ZNF598 shRNA upon UV irradiation have lower percentage of annexin V positive cells as compared to control cell expressing non-specific shRNA (Figure 5C).

In addition to conclusively show the involvement of ZNF598 overexpression in UV-induced apoptosis, we overexpressed ZNF598 in HCT116 cells and then measured the expression of UV-induced apoptotic genes. The results showed that ZNF598 over expression leads to increase in expression of UV-induced apoptotic genes and also increased apoptosis (Figure 5D and 5E). These results led us to conclude that loss of ZNF598 leads to inhibition of UV-induced apoptotic gene expression that mediates resistance to UV-induced apoptosis.

## DISCUSSION

**Figure 6:**
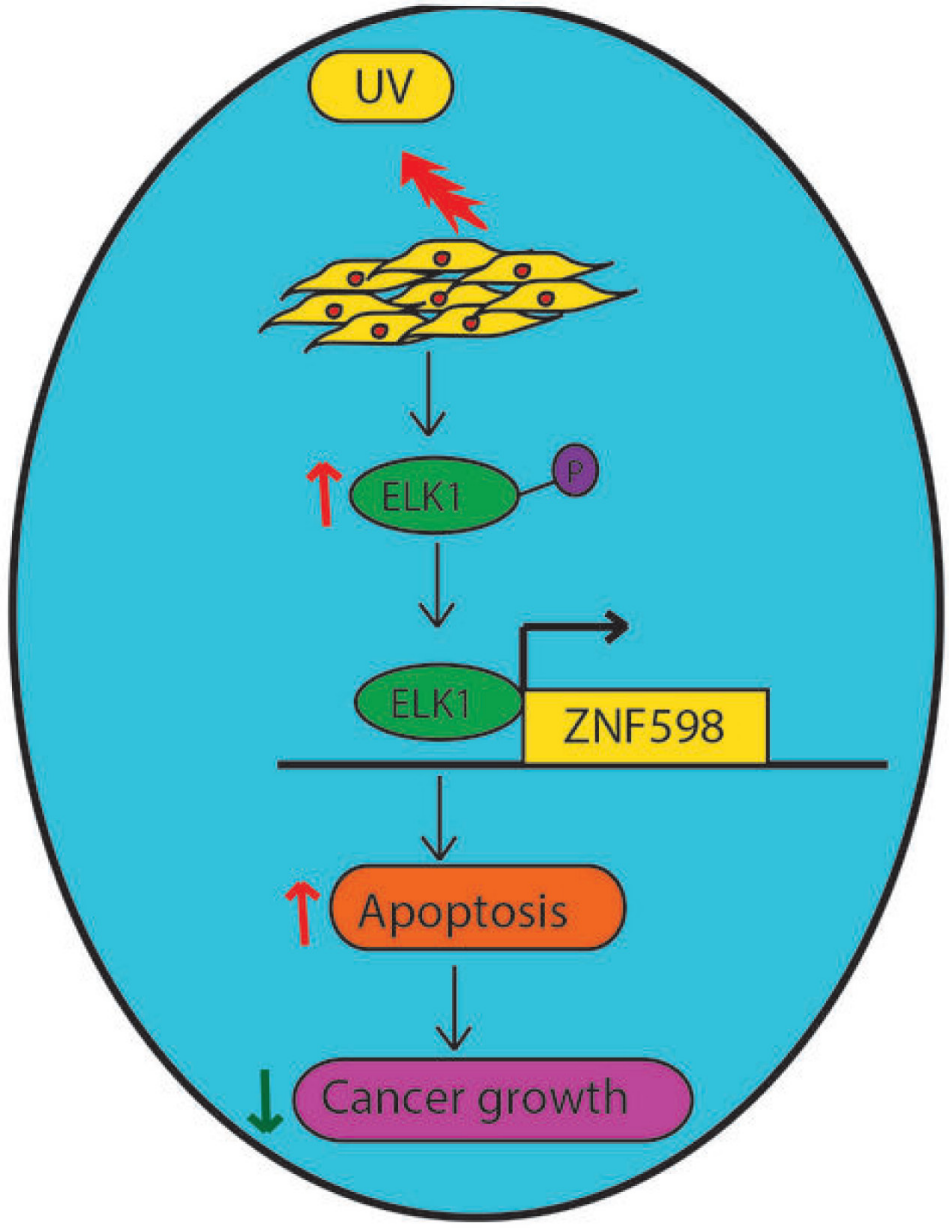
Model showing the role of ZNF598 in UV-induced apoptosis Upon UV irradiation, ELK1 gets upregulated, which then increases ZNF598 levels. ZNF598 by inducing the expression of UV-induced apoptotic genes promote apoptosis and thus preserves genome instability, and prevent cancer progression.

In this report, we identify a ZNF598 as key regulator of UV-induced apoptosis. An overview of our results is presented in Figure 6 and discussed below. First, we find upon UV irradiation ZNF598 expression is up regulated in multiple cancer cell lines. Additionally, ZNF598 knockdown in cancer cells leads to resistance to UV-induced apoptosis. Furthermore, we find both ELK1 mRNA level as well as phospho ELK1 level is upregulated upon UV irradiation, which leads to increase in ZNF598 expression. Cells expressing ELK1 shRNA are resistant to UV-induced apoptosis and phenocopy ZNF598 knockdown. Collectively, we demonstrate that loss of ZNF598 protects cancer cells from UV-induced apoptosis and is thus a new regulator of UV-induced apoptosis.

Previous literature on ZNF598 has pointed towards its association with ribosome and its role in regulating translation [19]. Studies in drosophila have shown that ZNF598 is a component of m4EHP and is involved in translation regulation [20]. It is required for ribosome stalling at poly (A) stretch of messenger RNA. And therefore, its loss leads to failed ribose stalling which eventually effects reading through of poly (A) altering protein localization and solubility. Studies have also illustrated that ZNF598 and RACK1 regulate Mammalian Ribosome-Associated Quality control function by 40S ribosome ubiquitination [21]. However, its role in mediating UV-induced apoptosis has never been shown. Our genome-wide shRNA screen performed with the aim to identify the factors that mediate UV-induced apoptosis led us to identify Zinc finger protein ZNF598 as a candidate that is involved in UV-induced apoptosis. Upon UV irradiation cells upregulate many DNA repair and apoptotic pathways, which is essential for maintaining genome integrity, preventing neoplastic transformation, and maintaining disease-free survival [22–27]. In our study, we find that cancer cells in response to UV irradiation upregulate ZNF598 expression. We also find that when ZNF598 is knocked down in cancer cells, they become resistant to UV-induced apoptosis. Additionally, we find that loss of ZNF598 promotes cancer cell survival upon UV irradiation. Next, we find that both phosphorylation and expression of transcription factor ELK1 just like ZNF598 is increased upon UV irradiation. ELK1 is a member of the Ets family of transcription factors and of the ternary complex factor (TCF) subfamily [28]. Previous studies have shown that TCF subfamily form a ternary complex by binding to the serum response factor and the serum response element in the promoter of the c-fos proto-oncogene. They also show that ELK1 is a nuclear target for the ras-raf-MAPK signaling cascade. Both UV irradiation and MEKK1 expression are important for JNK1 and JNK2 activation, which then leads to activation of TCK/ELK1 complex [28] and are consistent with our findings. Additionally, we observed that activated ELK1 transcriptionally upregulates ZNF598 levels and loss of ELK1 also resulted in resistance to UV-induced apoptosis, suggesting that similar to ZNF598, loss of ELK1 protects the cells against UV irradiation-induced apoptosis. In order to understand if ZNF598 effects the expression of genes involved in UV-induced apoptosis, we measured the expression of various UV-induced apoptotic genes like BAK and NOXA. Previous studies have shown that apoptotic genes like BAK and NOXA are induced upon UV irradiation [29–31]. We found that, cell expressing ZNF598 shRNA upon UV irradiation expresses lower levels of UV-induced apoptotic genes and have lower percentage of annexin V positive cells as compared to control cell expressing non-specific shRNA. In addition, ZNF598 overexpression result in up regulation of apoptotic gene expression and higher annexin V positive cells. These results led us to conclude that loss of ZNF598 mediates resistance to UV-induced apoptosis via regulating UV-induced apoptotic genes.

## MATERIALS AND METHODS

### Cell culture and plasmids

HCT116, H1299 and MCF7 were obtained from American Type Culture Collection (ATCC) and grown as recommended. ZNF598 was cloned in plasmid vector pEGFP-N1 expressing GFP tag.

### Transfections, shRNAs and preparation of lentiviral particles

*ZNF598, ELK, RNF17* and *KLHL5* and control non-specific shRNAs were obtained from OpenBiosystems. Table 3 shows the product IDs for all shRNAs. Lentiviral particles were prepared by co-transfecting the shRNA plasmids and lentiviral packaging plasmids, pSPAX2 and pMD2.G, into 293T cells using Effectene (Qiagen) and following the protocol at the Broad Institute’s website (http://www.broadinstitute.org/rnai/public/resources/protocols).

**Table 3:** Primer sequences for RT-qPCR analysis; clone ID and catalog numbers for shRNAs (Open Biosystems); antibodies used

Application	Gene symbol	Forward primer (5′-3′)	Reverse primer (5′-3′)
RT-qPCR	ZNF598	gccttccaggaggaggactt	ggggctgtgctaccttcttg
	ELK1	atctgtgacgctgtggcagt	ccagcttgaattcaccacca
	ACTIN	gccgggacctgactgactac	tcttctccagggaggagctg
	RNF17	aatgccttgccttgcagaat	agtgcctccatgctccattt
	KLHL5	ggaggaagcagtggatggac	gttccagagcagtgcattcg
	**Gene symbol**	**Clone ID**	**Catalog number**
shRNAs	ZNF598	TRCN0000073160	RHS3979-98493176
		TRCN0000073162	RHS3979-97061459
	ELK1	‘TRCN0000007450	‘RHS3979-9576412
		‘TRCN0000007453	RHS3979-9576415
	RNF17	TRCN0000062778	RHS3979-9629962
		TRCN0000062780	RHS3979-9629964
	KLHL5	TRCN0000116327	RHS3979-98058996
		TRCN0000116330	RHS3979-98058930
**Antibodies**	**Protein**	**Source**	**Dilution**
	ELK1	Cell signaling	1:1000 dilution
	Phospho-ELK1	Cell signaling	1:1000 dilution

### Antibodies and immunoblot analysis

Whole cell protein extracts were prepared using IP lysis buffer (Pierce) containing Protease Inhibitor Cocktail (Roche) and Phosphatase Inhibitor Cocktail (Sigma-Aldrich, St. Louis, MO). Protein concentration was estimated using a Bradford Assay kit (Bio-Rad). Proteins were resolved on 10% or 12% polyacrylamide gels and transferred to PVDF membranes using a wet transfer apparatus from Bio-rad. Membranes were blocked with 5% skim milk and probed with primary antibodies, followed by the appropriate secondary HRP-conjugated antibody (GE healthcare, UK). Blots were developed using the Supersignal Pico Reagent (Pierce). Information of the antibodies used in this study is provided in Table 3.

### UV irradiation, cell viability analysis, clonogenic assay and soft agar assay

Cells were UV-irradiated at various doses and time points, as indicated in the figures. To measure cell viability after irradiation, 0.2 × 10^6^ cells were plated in 6-well plates and UV-irradiated at different doses as indicated in the figures. At 48 hrs after irradiation, cells were harvested, mixed with an equal volume of trypan blue solution (Life Technologies), and counted using Countess (Life Technologies). The relative viability was plotted in reference to untreated cells.

The clonogenic ability of the cells stably expressing control or *ZNF598* shRNAs were measured in un-irradiated and UV irradiated conditions. For clonogenic assay, 5X10^5^ cells were seeded in a 6-well plate and 48 hrs after 20 J/m^2^ of UV irradiation 5 × 10^3^ cells were re-seeded in another 6 well plate. As an un-irradiated control, 5X10^3^ cells were seeded in a 6-well plate. After 2 weeks of plating, colonies were fixed with a fixing solution containing 50% methanol and 10% acetic acid and then stained with 0.05% Coomassie blue (Sigma-Aldrich). The relative number of colonies was calculated first by normalizing the average colony number for each sample under control un-irradiated condition and then plotting the average obtained from the triplicates carrying either indicated shRNAs against those with NS shRNA.

For the soft agar assay, individual cell lines were seeded in triplicate at three different dilutions, ranging from 5 × 10^3^ to 2 × 10^4^ cells. Cells were seeded into a 0.4% soft-agar layer. After 4–5 weeks images of the colonies were taken from inverted light microscope. Further colonies were stained with 0.005% crystal-violet solution and counted and the average area of each sample was calculated using Image J software and plotted. Each experiment was repeated at least twice.

### RNA isolation, RT-qPCR analysis

Total RNA was extracted using TRIzol (Life technologies), according to the manufacturer’s instructions. Total RNA was purified using RNAeasy mini columns (Qiagen). First-strand cDNA synthesis was performed using the ProtoScript M-MuLV First-Strand cDNA Synthesis Kit (New England Biolabs), and qPCR was performed using the Power SYBR Green PCR Master Mix (Applied Biosystems). The primers used for qPCR analysis are listed in Table 3. *Actin* mRNA was used to normalize RT-qPCR data.

### Chromatin immunoprecipitation

ZNF598 promoter sequence was downloaded from UCSC genome browser and analysed using rVisa 2.0. ChIP experiments were performed as described previously [32]. Normalized Ct (ΔCt) values were calculated by subtracting the Ct obtained with input DNA from that obtained with immunoprecipitated DNA (ΔCt = Ct(IP) − Ct(input)). Relative fold enrichment of a factor at the target site was then calculated using the formula 2^−(ΔCt(T)^^−^^ΔCt(Actb))^, where ΔCt(T) and ΔCt(Actb) are ΔCt values obtained using target and *Actb* (irrelevant) primers, respectively.

### Apoptosis measurement using annexin V staining

Cells were analyzed for apoptosis using Annexin V staining and flow cytometry using the FITC-Annexin Apoptosis Detection Kit I (BD Pharmingen), as per the manufacturer’s protocol.

### Statistical analysis

All experiments were performed at least three times in triplicate, and the data are expressed as mean ± standard error of the mean (SEM). The student’s *t*-test for two-tailed distribution with unequal variance was performed in Microsoft Excel to derive the *p*-values.

## SUPPLEMENTARY MATERIALS FIGURES



## Abbreviations

Abbreviation: Full name
CHIP: Chromatin Immuno-precipitation
shRNA: Short Hairpin RNA
mRNA: Messenger RNA
UV: Ultraviolet
MAPK: Map kinase
ROS: Reactive oxygen species
ZNF598: Zinc Finger protein 598
TCF: Ternary complex factor
