# Supplementary material for: Zinc finger protein 598 inhibits cell survival by promoting UV-induced apoptosis
Source: Oncotarget. 2017 Dec 23;9(5):5906–18. doi: 10.18632/oncotarget.23643 (PMC5814183; doi:10.18632/oncotarget.23643)
Supplement: Supplementary file 1 [file oncotarget-09-5906-s001.pdf]

Zinc finger protein 598 inhibits cell survival by promoting UV-induced apoptosis

SUPPLEMENTARY MATERIALS

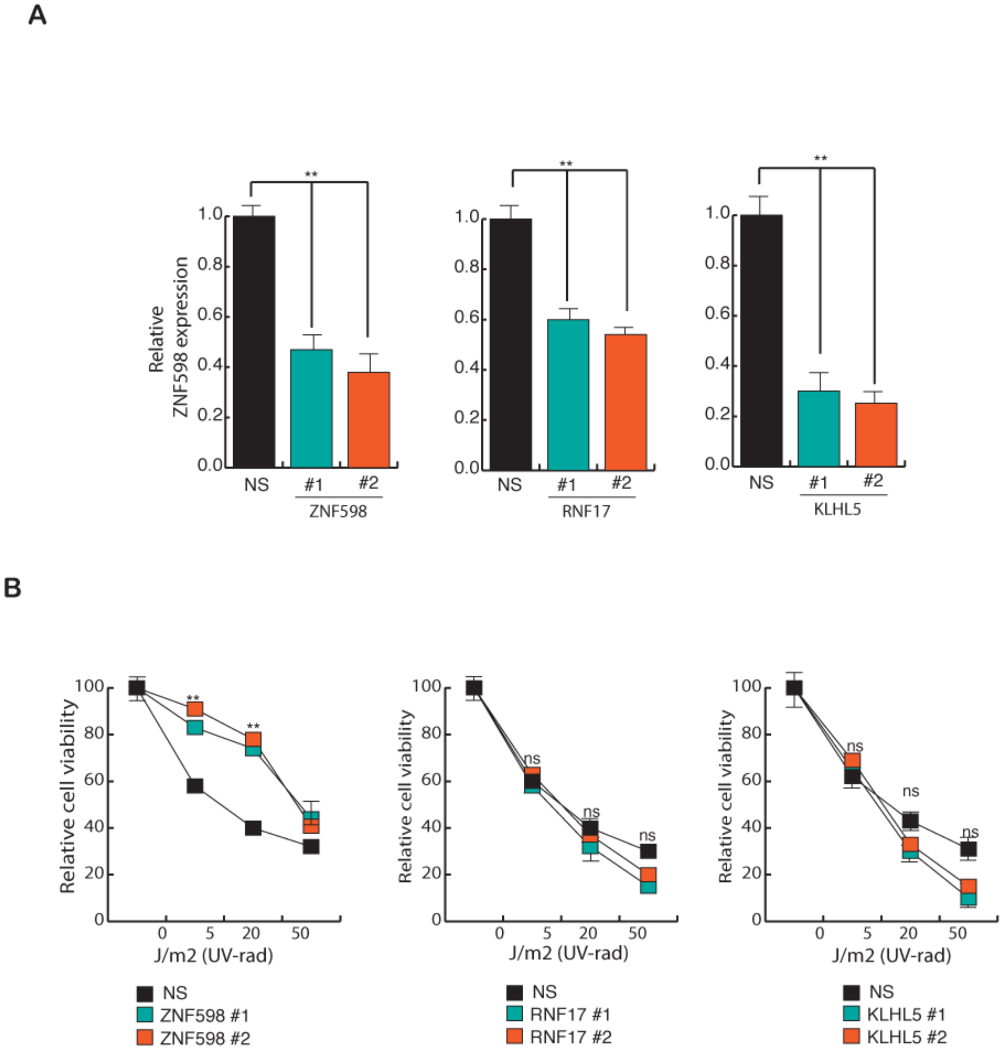

Supplementary Figure 1: ZNF598 inhibition leads to resistance to UV-induced apoptosis. (**A**) Relative mRNA expression of the genes (ZNF598, RNF17 and KLHL5) in HCT116 cell expressing NS shRNA and gene specific shRNA. Relative gene expression is plotted with respect to control cell expressing non-specific shRNA. (**B**) HCT116 cell expressing either non-specific shRNA or ZNF598, RNF17 and KLHL5 shRNA were UV irradiated at the indicated doses, and cell viability was measured by the trypan blue exclusion assay 48 hours post-irradiation. The cell viability relative to the un-irradiated control is plotted. Error bar shows Standard Error Mean (SEM). ($^{**}p < 0.001$, ns, not significant).

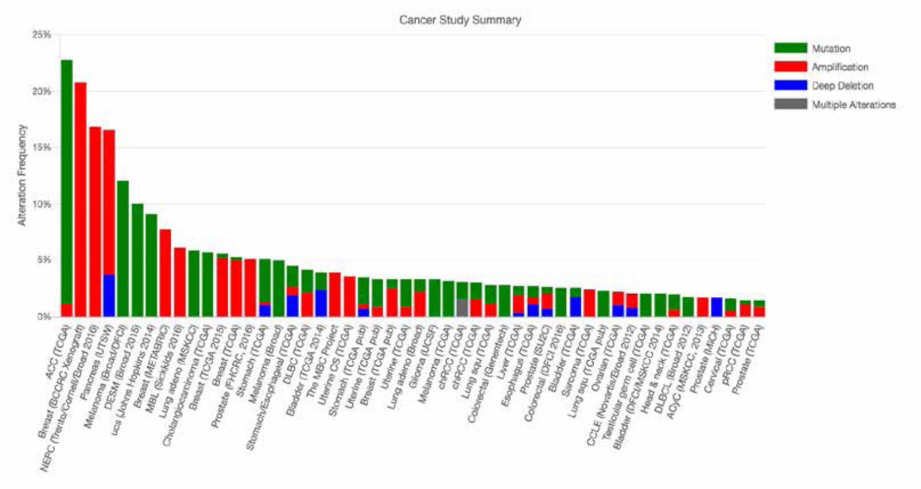

Supplementary Figure 2: ZNF598 gene carries significant inactivating mutation in various cancer type (A) Publicly available cBioportal database was analyzed for ZNF598 mutation and Copy number alteration for different cancer type.

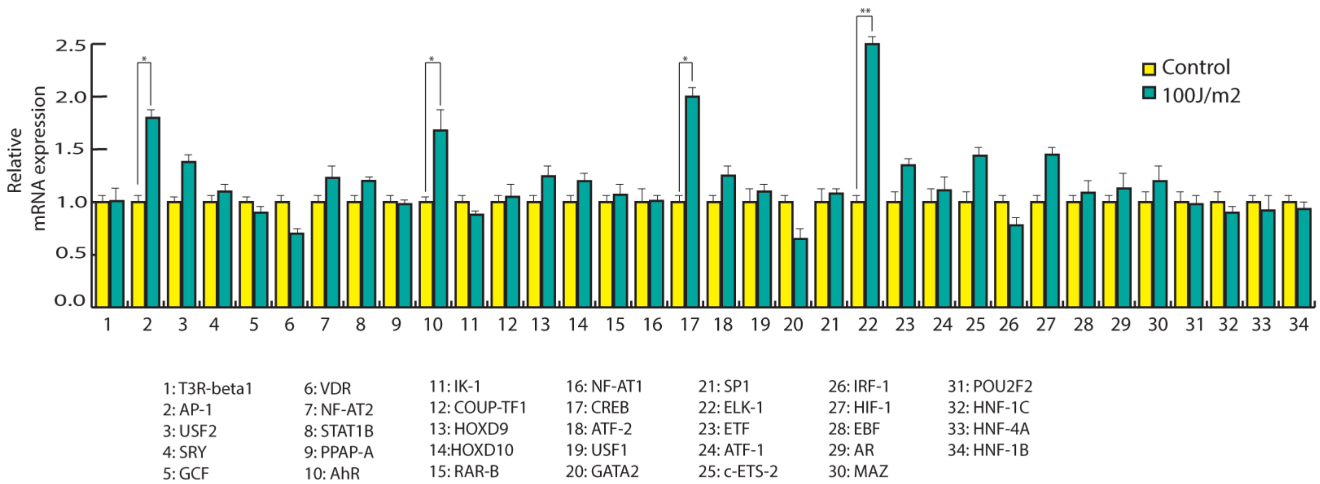

Supplementary Figure 3: UV irradiation significantly alters ELK1 mRNA expression (A) HCT116 cells were UV irradiated at 100 J/m^2 and mRNA expression via qRT-PCR was measured 24 hours after irradiation. Relative gene expression is plotted with respect to control un-irradiated cell.

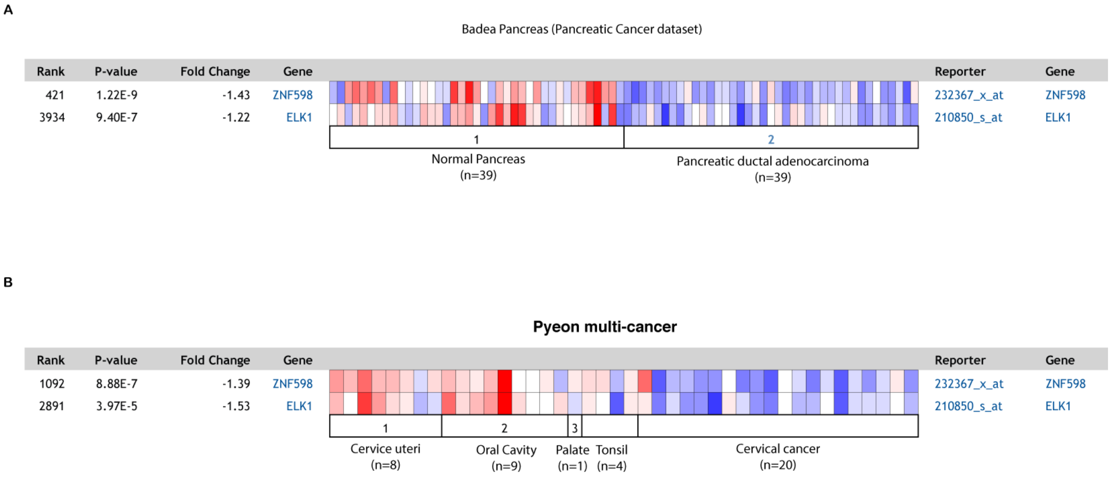

Supplementary Figure 4: ELK1 expression positively correlates with ZNF598 expression (**A**, **B**) Publicly available Oncomine database was analyzed for both ELK1 and ZNF598 expression for different cancer type. Co-expression of ELK1 and ZNF598 is plotted.
